# Differential Diagnosis between Osteo Porosis and Rheumatism in Horses*Read before the New Jersey State Veterinary Society.

**Published:** 1893-03

**Authors:** James D. Hopkins


### DIFFERENTIAL DIAGNOSIS BETWEEN OSTEO POROSIS AND RHEUMATISM IN HORSES.*

By James D. Hopkins, V. S.

During a practice of twenty-one years I have met many veterinary practitioners, on cases where the ailment presented symptoms which made it exceedingly difficult to make a differential diagnosis, unless careful attention is given to the history of the case, and a complete knowledge of the peculiarities of both diseases are carefully considered.

About 18 months ago I was called on to examine a black mare, 6 years old, and could find no cause of unsoundness. After every drive of any distance this mare showed lameness, sometimes in front, at other times in the hind extremities, which under appropriate treatment disappeared.

The owner, becoming tired of the continued annoyance, another veterinarian was called, who pronounced the disease rheumatism. This mare did not present any external evidence of osteo porosis. There was no enlargement of the bones of the face or lower jaw. In the joints, where the lameness occurred, there was no swelling; there was heat, but not painful on manipulation. The distance driven determined the amount of lameness, either light or excessive. Her appetite was poor, pulse and temperature elevated.

Six months later this mare, through a change in owners, again

*Read before the New Jersey State Veterinary Society.

came under my observation; and now the external evidence of osteo porosis was very plain in the enlargement of the bones of the face and lower jaw. My treatment of this mare was mineral tonics, diuretics, and local applications to affected joints.

Another case, worthy of mention, was a grey draft horse, 8 years old, who, while standing with his mate hitched to a heavy truck, grasped with his teeth the pole of the wagon with such force that the three upper incisors on the right side were turned up and outward.

This horse had been gradually losing his gait; appetite poor; but had been kept at his work. He was in good condition; pulse normal; temperature, 100½ Farh. The ramus of the lower jaw was fully two inches in diameter; the bones of the face much enlarged; but, being a healthy horse, the owner supposed it was natural.

This animal was not treated, as I thought the case hopeless, and he gradually declined until, about six months later, when from the ravages of disease, emaciation, and loss of strength, he was unable to arise from the recumbent position without help, the owner destroyed him.

Osteo porosis is an insidious disease, progressing slowly but surely, and generally the first thing noticed is the lameness. This will be recurrent; also metastatic in character. Sometimes the lameness will be slight, again very severe. The veterinarian will be at a loss to account for it, unless he examines the animal for constitutional disease. The horse may for a long time perform his daily task, and look thrifty unless exposed to hardship. In some cases there is a gradual loss of gait—stiff when compelled to turn short around; capricious appetite; unthrifty appearance of the coat; easily fatigued; the bones of the face and lower jaw are enlarged; pulse and temperature is elevated; grave changes take place in the integrity of osseous structure, and, with the constitutional disturbance, emaciation rapidly follows, and the end is soon brought about through exhaustion of the vital forces, unless the owner humanely ends the suffering.

This disease affects all classes of horses—the carriage horse as well as those used for draft; among horses kept in palatial stables, where every attention is given to sewerage and ventilation, and fed on the best food obtainable, as well as among those that are poorly housed and kept.

Some years ago, when this disease was first observed among the horses of this country, it was attributed to their feeding on hard corn, and was named "Big Head !" It has been demonstrated that cattle and sheep are also great sufferers from this disease.

The treatment of osteo porosis is very unsatisfactory. Remedial agents seem to be futile. If the horse, in the early stage of the disease, can be turned to fresh grass for a few months, he will in most cases return in fair condition, and do service for a long time.

For the morbid anatomy of osteo porosis, I refer you to Prof. Williams' "Principles and Practice of Veterinary Surgery," page 184.

Prof. Robertson, in his "Practice of Equine Medicine," gives an excellent account of the rheumatic form certain cases of influenza assume :

"In influenza our patients are at times afflicted with a rheumatic complication, in which the serous and fibro-serous textures of joints and tendons are swollen—hot, tense and tender on manipulation; the lameness is great, and the temperature, pulse and respiration increased. This condition is metastatic, attacking in some instances all the limbs in the course of a few days. This disease readily yields to remedial agents, the patient making a good recovery." Quite a difference from the insidious ravages of osteo porosis !

Again on page 238, Prof. Robertson describes acute rheumatism as attacking the same tissues as in the above form, admits of its heredity, and spontaneous origin when the animals are exposed to certain atmospherical and telluric influences ; and he also admits that young stock are more frequently affected than adults.

The joints and other situations of the limbs, which are the seat of local inflammatory action, are swollen, hot and tense; also capsules of joints and sheaths of tendons. There is a deranged condition of the intestinal canal, and the usual elevation of temperature, with a frequent, full and unyielding pulse. The extension of the inflammatory action to the heart and its appendages, give rise to another series of symptoms, as restlessness, anxious expression of countenance, sometmies a cough, difficulty in respiration, and palpitation when caused to move rapidly.

But the lameness is the attractive symptom, and it is sometimes severe and sudden.–it may affect one limb or two at the same time. Swelling of the affected member does not seem to give relief from the pain,which is so great, that when the animal is forced to move, it causes the animal to perspire freely.

Prof. Robertson and Prof. Williams, both standard authorities on veterinay matters, state that rheumatism generally affects growing stock, when they are exposed to inclement weather, or housed in damp stables, or raised on low lying farms; and rarely affects adult horses, except as a sequel to some inflammatory disease.

Now, gentlemen, after mature consideration of the differential symptoms presented in the horse, when suffering from osteo porosis, and the descriptions of symptoms of rheumatism, as furnished by standard authorities, and after a practice of twenty-one years among horses, I am obliged to confess that I have never seen a case of rheumatism in the adult horse, except as a sequel to some febrile complaint, in the form of rheumatic arthrites.

